# Supplementary material for: Basal Values of Biochemical and Hematological Parameters in Elite Athletes
Source: Int J Environ Res Public Health. 2022 Mar 5;19(5):3059. doi: 10.3390/ijerph19053059 (PMC8910271; doi:10.3390/ijerph19053059)
Supplement: Supplementary file 1 [file ijerph-19-03059-s001.zip › ijerph-1587447-supplementary.pdf]

Table S1. List of sport federations included in this study.

Federations	Male			Female		
	Samples	Athletes	Age	Samples	Athletes	Age
Athletics	1871	318	25.18 ± 6.35	1396	189	25.12 ± 5.46
Badminton	127	25	21.10 ± 4.38	98	10	21.07 ± 2.56
Basketball	564	250	27.13 ± 9.17	251	125	23.83 ± 5.67
Handball	38	29	29.55 ± 5.59	87	75	22.34 ± 5.56
Boxing	246	55	24.82 ± 8.37	56	26	25.41 ± 3.83
Cycling	272	158	27.39 ± 7.05	48	26	27.00 ± 6.58
Orienteering sport	125	38	24.35 ± 6.98	41	21	21.34 ± 6.51
Paralympic Sports	686	201	29.56 ± 8.92	236	48	25.62 ± 7.47
Winter sports	165	44	22.38 ± 5.08	75	20	22.33 ± 4.72
Fencing	75	39	21.95 ± 4.58	56	25	21.21 ± 2.48
Football	133	95	29.66 ± 7.64	30	29	24.70 ± 4.44
Gymnastics	145	28	23.37 ± 6.97	164	49	21.06 ± 5.21
Golf	46	21	30.57 ± 15.04	34	20	20.62 ± 4.92
Weightlifting	171	22	22.06 ± 2.85	179	15	23.08 ± 4.22
Grass hockey	84	51	24.61 ± 5.09	345	56	24.00 ± 3.77
Judo	224	71	23.68 ± 5.13	162	58	24.28 ± 5.79
Karate	94	28	23.52 ± 5.34	65	20	23.68 ± 5.91
Olympic fight	119	46	22.29 ± 4.54	57	17	22.81 ± 3.93
Mountaineering	72	44	26.07 ± 8.74	42	26	27.14 ± 5.64
Motorcycling	5	5	19.40 ± 2.19	1	1	20 (*)
Swimming	636	168	22.14 ± 5.64	458	128	22.42 ± 4.11
Canoeing	921	97	23.41 ± 5.11	378	61	22.09 ± 4.00
Rowing	337	69	24.78 ± 5.03	120	39	25.53 ± 5.43
Rugby	194	69	25.93 ± 3.91	589	98	24.98 ± 4.71
Taekwondo	159	25	23.16 ± 4.50	48	12	24.96 ± 6.34
Tennis	1	1	21.00 (*)	3	2	21.67 ± 3.21
Table tennis	56	20	21.80 ± 4.29	2	1	25.00 ± 0.00
Archery	48	16	21.71 ± 2.67	42	14	20.36 ± 2.06
Olympic shot	59	24	26.14 ± 6.08	59	18	23.69 ± 6.06
Triathlon	535	93	23.23 ± 5.70	317	43	25.79 ± 7.64
Sailing	101	45	28.77 ± 6.02	74	25	26.95 ± 5.21
Volleyball	143	63	25.15 ± 4.15	45	33	24.57 ± 4.93
**Total**	**8452**	**2258**	**24.93 ± 6.87**	**5558**	**1330**	**24.13 ± 5.49**

(*) Only one data.

Table S2. Analytical variation coefficient of biochemical parameters analyzed on Beckman AU400 clinical chemistry analyzer.

	**Analytical CV (%)**
Glucose (Glc)	2.59
Urea	2.01
Creatinine (CREA)	1.66
Uric acid (UA)	1.53
Total cholesterol (CHOL)	2.13
Triglycerides (TG)	3.26
Total protein (TP)	2.28
Albumin (Alb)	2.74
Creatine kinase (CK)	2.24
Aspartate aminotransferase (AST)	2.02
Alanine aminotransferase (ALT)	1.62
Gamma-glutamyltransferase (GGT)	1.79
Lactate dehydrogenase (LDH)	2.60
Sodium (Na)	0.59
Potassium (K)	1.26
Chloride (Cl)	0.66
Calcium (Ca)	2.73
Inorganic Phosphate (Phos)	1.92
Magnesium (Mg)	2.75
Iron (Fe)	3.16
Total bilirubin (TBIL)	2.13

Table S3. Analytical variation coefficient of hematological parameters analyzed on Siemens Advia 120 hematological analyzer.

	**Analytical CV (%)**
Erythrocytes (RBC)	1.05
Hemoglobin (Hb)	0.90
Hematocrit (Hct)	1.24
Mean Cell Volume (MCV)	0.67
Mean Cell hemoglobin (MCH)	1.41
Mean Cell hemoglobin concentration (MCHC)	1.52
Red cell distribution wide (RDW)	1.09
Hemoglobin distribution wide (HbDW)	1.90
Total leukocytes (WBC)	2.34
Lymphocytes	3.46
Neutrophils	2.06
Monocytes	8.00
Eosinophils	11.18
Basophils	24.18
Large unstained cells (LUC)	16.93
Platelets (Plt)	3.13
Mean platelet volume (MPV)	1.86
Plateletcrit (Pct)	3.83
Platelet volume distribution width (PDW)	5.18
Reticulocyte count (Rtc)	10.03
Reticulocyte percentage (Rtc%)	9.73
Mean reticulocyte volume (MCVr)	1.35
Mean reticulocyte corpuscular hemoglobin (MCHr)	0.77
High fluorescence reticulocyte population (RETH)	38.52
Medium fluorescence reticulocyte population (RETM)	15.33
Low fluorescence reticulocyte population (RETL)	2.51
